# Protection against Ischemic Heart Disease: A Joint Role for eNOS and the K_ATP_ Channel

**DOI:** 10.3390/ijms24097927

**Published:** 2023-04-27

**Authors:** Paolo Severino, Andrea D’Amato, Massimo Mancone, Alberto Palazzuoli, Marco Valerio Mariani, Silvia Prosperi, Vincenzo Myftari, Carlo Lavalle, Giovanni Battista Forleo, Lucia Ilaria Birtolo, Viviana Caputo, Fabio Miraldi, Cristina Chimenti, Roberto Badagliacca, Viviana Maestrini, Raffaele Palmirotta, Carmine Dario Vizza, Francesco Fedele

**Affiliations:** 1 Department of Clinical, Internal, Anesthesiology and Cardiovascular Sciences, Sapienza University of Rome, Viale del Policlinico 155, 00161 Rome, Italy; 2 Cardiovascular Diseases Unit, Cardio Thoracic and Vascular Department, Le Scotte Hospital, University of Siena, 53100 Siena, Italy; 3 Department of Cardiology, Luigi Sacco University Hospital, 20157 Milan, Italy; 4 Department of Experimental Medicine, Sapienza University of Rome, 00161 Rome, Italy; 5 Interdisciplinary Department of Medicine, University of Bari ‘Aldo Moro’, 70121 Bari, Italy

**Keywords:** coronary blood flow, ischemic heart disease, microvascular dysfunction, ATP sensitive potassium channels, endothelial nitric oxide synthase, genetic polymorphism

## Abstract

Genetic susceptibility may influence ischemic heart disease (IHD) predisposition and affect coronary blood flow (CBF) regulation mechanisms. The aim of this study was to investigate the association among single nucleotide polymorphisms (SNPs) of genes encoding for proteins involved in CBF regulation and IHD. A total of 468 consecutive patients were enrolled and divided into three groups according to coronary angiography and intracoronary functional tests results: G1, patients with coronary artery disease (CAD); G2, patients with coronary microvascular dysfunction (CMD); and G3, patients with angiographic and functionally normal coronary arteries. A genetic analysis of the SNPs rs5215 of the potassium inwardly rectifying channel subfamily J member 11 (KCNJ11) gene and rs1799983 of the nitric oxide synthase 3 (NOS3) gene, respectively encoding for the Kir6.2 subunit of ATP sensitive potassium (K_ATP_) channels and nitric oxide synthase (eNOS), was performed on peripheral whole blood samples. A significant association of rs5215_G/G of KCNJ11 and rs1799983_T/T of NOS3 genes was detected in healthy controls compared with CAD and CMD patients. Based on univariable and multivariable analyses, the co-presence of rs5215_G/G of KCNJ11 and rs1799983_T/T of NOS3 may represent an independent protective factor against IHD, regardless of cardiovascular risk factors. This study supports the hypothesis that SNP association may influence the crosstalk between eNOS and the K_ATP_ channel that provides a potential protective effect against IHD.

## 1. Introduction

Ischemic heart disease (IHD) is the leading cause of mortality worldwide [1,2,3,4]. Several pathological conditions, in particular diabetes mellitus, arterial hypertension, dyslipidemia, smoking habit, obesity, and age are already recognized as major cardiovascular (CV) risk factors [3]. To reduce major cardiovascular events (MACE), guidelines [1,2,3,4], based on large randomized controlled trials, are even more stringent regarding the control of CV risk factors, pursuing the achievement of even more severe targets. Commonly, the term IHD is used as synonymous with atherosclerotic disease [4]. However, patients with coronary atherosclerotic disease may not develop signs and symptoms of myocardial ischemia, while myocardial ischemia may also occur in patients without obstructive epicardial atherosclerotic disease. In this condition, other pathophysiological mechanisms, such as coronary microvascular dysfunction (CMD), are responsible for myocardial ischemia [5,6].

CMD defines a set of structural and functional abnormalities involving the coronary microvasculature that results in inadequate coronary blood supply [5,6,7]. CMD is present in approximately half the patients with non-obstructive coronary artery disease [8]. However, CMD and epicardial coronary atherosclerosis are closely related each other: the increased microvascular resistances, observed in CMD, slow the upstream blood flow with consequent storage of inflammation and oxidative stress (OS) mediators, which promote atherosclerotic process [9,10,11]. On the other hand, atherosclerosis and concurrent comorbidities, such as hyperglycemia, hyperlipidemia, inflammation, and OS promote the imbalance of proteins involved in coronary blood flow (CBF) regulation, predisposing patients to CMD [9,10,11].

CV events may also occur in the absence of CV risk factors. In this regard, a role for genetic susceptibility to IHD, acting independently of classical CV risk factors, has been postulated [12,13,14]. Genetic predisposition may influence IHD susceptibility at different levels, influencing the atherosclerotic process, local and systemic inflammation, OS, and CBF regulation mechanisms [12,13,14,15]. Genetic susceptibility is often considered a highly negative factor that predisposes patients to myocardial ischemia and worse outcomes; however, some evidence of a protective role carried out by genetic variants has been described [16,17,18,19]. In particular, several single nucleotide polymorphisms (SNPs) have been associated with IHD [12,13,14,15,16,17,18,19].

The coronary arterial tree consists of a vessels network with different sizes and functions that are always in connection with each other. Epicardial coronary arteries have a conductance role offering minimal resistance to CBF [20,21]. Pre-arterioles, arterioles, and capillaries represent the coronary microcirculation, which is the main site of coronary vascular resistance and CBF regulation. Epicardial and microvascular districts establish a complex network which has the role of guaranteeing an adequate CBF, according to cardiomyocytes’ metabolic demand [20,21]. Even though the real role of the mechanisms responsible for the crosstalk between coronary flow and myocardial metabolism has been not clearly identified yet, it is known that, in physiological conditions, CBF regulation is mediated by various regulatory systems, including endothelial, neurohumoral, nervous, metabolic, and myogenic mechanisms [20,21,22]. These regulatory mechanisms include several pathways in which many bioactive molecules interact with each other, like small pieces of a complex puzzle.

Among them, endothelial function and the nitric oxide (NO) pathway are crucial in several physiological processes. The NO pathway is involved in vasodilation, platelet aggregation inhibition, counteracting inflammation, and OS [7,8,10]. The impairment of the NO pathway has been already associated with IHD, and it can be hypothesized that certain genetic variants encoding for proteins involved in the NO pathway may influence NO production both in quantitative and qualitative terms. In addition, coronary ion channels present on endothelial and vascular smooth muscle cells (VSMCs) are the end effectors of CBF regulation mechanisms [16,20,21,22]. Through regulation of the intra- and extracellular concentration of main ions, such as potassium (K^+^), sodium (Na^+^), calcium (Ca^++^) and magnesium (Mg^++^), these ion channels are involved in several physiological processes, such as paracrine molecule secretion, endothelial function, and vasomotor tone regulation [16,20,21,22].

We hypothesized that genetic susceptibility may influence the predisposition to IHD, primarily through the regulation of CBF. The aim of the present study is to investigate the association among SNPs of genes encoding for proteins involved in CBF regulation and IHD. In particular, we focus on the interaction among the genetic variants of endothelial nitric oxide synthase (eNOS) and adenosine triphosphate sensitive potassium (K_ATP_) channels genes and IHD susceptibility.

## 2. Results

In the present study, a total of 468 patients with indication to perform coronary angiogram (CAG) due to suspected and/or documented acute and/or chronic myocardial ischemia were consecutive enrolled. The mean age was 68 [57.7;75] years and 67.7% (*n* = 313) of the study population was represented by males. The most common CV risk factor was arterial hypertension (88.5%, *n* = 409), followed by dyslipidemia (52%, *n* = 240) and smoking habit (44.6%, *n* = 206). Ischemic alterations at electrocardiogram (EKG) were recorded in 78% (*n* = 360) of the population, while the mean left ventricular ejection fraction (LVEF) was 50% [40;55]. A total of 71% (*n* = 328) of the population was affected by CAD and included in G1, while 14.7% (*n* = 68) was affected by CMD and included in G2. Among the CMD patients, 29% (*n* = 20) had endothelial-dependent CMD, while 1.5% (*n* = 1) had endothelial-independent CMD and 70% (*n* = 47) had both endothelial-dependent and independent CMD. A total of 14.3% (*n* = 66) of the total population was healthy, with angiographic and functionally normal coronary arteries, and they were included in G3. Baseline features and differences according to the three groups are presented in Table 1.

Table 2 lists the results of gene analysis for the SNPs rs5215 of the potassium inwardly rectifying channel subfamily J member 11 (KCNJ11) gene encoding for the subunit inward-rectifier potassium channels (Kir6.2) of the ATP sensitive potassium (K_ATP_) channel, and rs1799983 of the nitric oxide synthase 3 (NOS3) gene encoding for the endothelial nitric oxide synthase (eNOS), as well as their interactions. The co-presence of rs5215_G/G, where G defines guanin, of KCNJ11, and rs1799983_T/T, where T defines thymine, of NOS3 was significantly different among the three groups (*p* = 0.008). At post-hoc analysis, this SNPs association is significantly more abundantly detected in the G3 group compared with the G1 and G2 groups (G1-G2: *p* = 0.246; G1-G3: *p* = 0.026; G2-G3: *p* = 0.019). Despite the SNP rs1799983_G/T of NOS3 not reaching statistical significance (*p* = 0.052), at post-hoc analysis, this SNP is significantly more expressed in G2 compared with G3 (G1-G2: *p* = 0.126; G1-G3: *p* = 0.209; G2-G3: *p* = 0.018).

Univariable and multivariable models of the logistic regression analysis were performed to study predictors for CAD, CMD, and IHD (CAD+CMD), and they have been included in Table 3, Table 4 and Table 5 respectively. In the multivariable analysis, the co-presence of the SNPs rs5215_G/G and rs1799983_T/T of KCNJ11 and NOS3 respectively was confirmed to represent an independent protective factor against IHD (OR: 0.185; 95%CI: 0.440–0.770; *p* = 0.020).

## 3. Discussion

IHD is the most frequent cause of mortality and morbidity worldwide, predisposing patients to several sequelae, such as heart failure [1,2,3,4,23]. IHD has a pathophysiology that reflects the complexity of coronary circulation, characterized by several vascular districts absolving to different functions with the final goal of guaranteeing an adequate blood flow, according to the myocardial metabolic demand. Despite coronary epicardial arteries and coronary microcirculation having different functions, and regulatory mechanisms acting differently, according to the considered district, they are closely interconnected [16,20]. IHD is often considered synonymous with atherosclerotic disease of epicardial coronary arteries. However, several studies underline the role of CMD in determining myocardial ischemia, regardless of obstructive atherosclerotic plaque presence [6,7,8,9,10,16]. CMD and CAD may often coexist for different reasons: (i) atherosclerosis promotes CMD, and they may be the result of CV risk factor actions at the coronary level. Hyperglycemia, dyslipidemia, inflammation, shear stress, and OS contribute to atherosclerosis onset and progression, as well as imbalance of regulatory CBF mechanisms, at the microvascular level, the main site of coronary resistance regulation; (ii) a primary CMD and CBF regulatory mechanism imbalance is responsible for hemodynamic alteration at the epicardial level, favoring storage of oxidized LDL, inflammation, and OS products, and promoting atherosclerotic disease [6,7,8,9,10].

Regulatory mechanisms of CBF may play a crucial role in maintaining the balance of the crosstalk between coronary circulation and cardiomyocytes [6,7,8,9,10,16,20]. For this reason, a primary dysfunction of CBF regulatory mechanisms may directly be associated with myocardial ischemia. Several pathways and many molecules are involved in the regulation of CBF, and ion channels are the end effectors of these fine mechanisms. These proteins work together and influence each other with repercussions on CBF and myocardial ischemia susceptibility [18,19,24,25,26].

The NO pathway and endothelial function are involved in several physiological mechanisms, assuming a central role in the crosstalk between coronary circulation and cardiomyocyte metabolism [27,28]. NO promotes vasodilation, and it counteracts platelet aggregation, OS, inflammation, and VSMCs proliferation. For this reason, endothelial dysfunction may have a central role in the susceptibility to myocardial ischemia, including a wide spectrum of pathological processes such as plaque instability, inflammation, microvascular hyperpermeability, and vasomotor tone dysregulation [27,28]. According to NO effects, the assumption that some genetic variants of proteins involved in the NO pathway may assume a predisposing or protective role against myocardial ischemia is also justifiable.

In addition, coronary potassium channels, in particular K_ATP_, may play a central role in coronary physiology. They are expressed by the endothelium and VSMCs and are involved in cell hyperpolarization and coronary vasodilation [29,30,31]. K_ATP_ channels regulate the intracellular Ca^++^ concentration and NO production by the endothelium [32,33,34,35]. For this reason, K_ATP_ channel activity and NO production may be related to each other, resulting in tangible hemodynamic effects.

While it is not completely understood how CBF regulation mechanisms work and interact among them, we hypothesize that the interplay between the NO pathway and the K_ATP_ channel may influence the predisposition to IHD, affecting the crosstalk between coronary circulation and cardiomyocyte metabolism. This interaction has been already described in other tissues and with contrasting results. Schrage et al. [36] demonstrated that in a subgroup of patients, the inhibition of NO, K_ATP_, and prostaglandins was associated with a strong reduction in skeletal muscle blood flow, and K_ATP_ may acquire more importance in blood flow regulation when NO and prostaglandin pathways are inhibited. Merkus et al. [37] observed that during inhibition of NO, K_ATP_, and adenosine, there were not significant abnormalities in CBF. The inhibition of coronary K_ATP_ was associated with reduced CBF, both at rest and during exercise, and myocardial ischemia [37]. Ishibashi et al. [38] demonstrated that the opening of K_ATP_ channels was the main mechanism of coronary vasodilation; when these were inhibited, NO and adenosine played a main role in replacing their function, increasing CBF in response to exercise. Hein et al. [39] demonstrated an interaction between K_ATP_ and NO in determining coronary vasodilation. Low-dose adenosine causes the opening of endothelial K_ATP_ channels, which subsequently causes NO production and release and smooth muscle dilation. High doses of adenosine act directly on smooth muscle K_ATP_ channels causing consequent cell hyperpolarization. In the latter case, K_ATP_ may overcome NO’s role in coronary vasodilation [39]. Fujii et al. [40] demonstrated the interplay between the K_ATP_ channel and the NO pathway in cutaneous vasodilation. In particular, NO activated K_ATP_ channels on VSMCs, inducing vasodilation. It is interesting to note that there was a NO threshold beyond which K_ATP_ channels were activated by NO. Moreover, the activation of NO pathway induced by K_ATP_ opening has been also described [41]. This may allow the speculation that the final effect of an NO–K_ATP_ interaction may depend on the basal activity of each protein, which may be genetically influenced by SNPs of their encoding genes. Other interactions among potassium channels and the NO pathway in determining vasodilation in body districts other than coronary circulation have been described [42,43].

Genetics may play a primary role in defining the susceptibility to IHD. In fact, several SNPs of proteins involved in CBF regulation mechanisms, in particular ion channels and eNOS, have been investigated, attracting great attention, because they can be associated with myocardial ischemia susceptibility, regardless of CV risk factors [18,19,24,25,26,29,30,31]. Genetic has demonstrated a role of *primum movens* in CAD and CV risk factor occurrence. Many genetic loci are significantly associated with CAD. Genome studies usually focus on SNPs, even though many of them fail to reach the significance threshold or are in non-coding and intergenic regions with unknown functional significance. What is more, SNPs partially contribute to CAD pathogenesis; in fact, SNP-based genetic scores may represent an adjunctive weapon to prevent IHD [44,45].

We had previously already demonstrated a role for the KCNJ11 and NOS3 gene SNPs in IHD susceptibility [18,19]. KCNJ11, located on chromosome 11p15.1, catalyzes the synthesis of the Kir6.2 subunit of the K_ATP_ channel on endothelial and VSMCs [46]. Previous studies have shown its role as an independent protective factor for IHD, especially the SNP rs5215_G/G, which causes the substitution of valine–isoleucine at exon 1009 (ATC–GTC), possibly leading to a gain of function of K_ATP_ channels resulting in increased vasodilation [18,19]. The NOS gene is located on chromosome 7q35-7q36 [47], and it is responsible for the synthesis of NO through a catabolic reaction when L-arginine is available [28,48]. It is one of the most investigated proteins in IHD susceptibility. It consists of three different isoforms: neuronal isoforms (nNOS), inducible isoform (iNOS), and eNOS. The latter, which is also referred to as NOS3, plays an important role in the regulation of vasodilation and the mediation of blood pressure and blood flow by producing vasoactive molecules. Thus, it is reasonable to associate its expression with endothelial disfunction and myocardial ischemia, as it affects both the microcirculation and the epicardial arteries [28,49]. It has been speculated that eNOS SNPs may entail myocardial ischemia susceptibility through endothelial dysfunction. However, the current state-of-the-art studies do not always show significant results. More precisely, the gene allelic variants -786TC, -922AG and -1468TA have been proven to impact the pathogenetic mechanisms of myocardial infarction, increasing their action when associated with cigarette smoking [50]. The polymorphism rs2070744 was associated with myocardial infarction [51]. The SNP rs1799983_G/T, determining the substitution of a guanine (G) with a thymine (T) at position 894 in exon 7, results in the aminoacidic change at position 298 from glutamic acid to aspartic acid [18,52]. The final effect is reduced eNOS expression and NO production. The latter SNP represents an independent risk factor for IHD and acute coronary syndrome [52]. The association between SNP rs1799983 of NOS3 with IHD and CV risk factors has been demonstrated in several populations with different ethnicities [53,54].

Given the role of eNOS and the K_ATP_ channel in the fine regulation of CBF, as well as the evidence of the potential primary role of genetic susceptibility in IHD, we hypothesize that SNPs of the NOS3 and KCNJ11 genes may potentiate the interaction between eNOS and the K_ATP_ channel, exploiting an evident effect on CBF regulation and a subsequent hemodynamic effect, influencing the predisposition to IHD. In particular, among the SNPs analyzed, our results suggest that the co-presence of the SNPs rs5215_G/G of KCNJ11 and rs1799983_T/T of NOS3 is higher in healthy patients (G3) compared with CAD (G1) and CMD (G2) patients. It is interesting to notice that in our population, the presence of rs5215_G/G of KCNJ11 or rs1799983_T/T of NOS3 alone did not reach a significant protective role, while the association of these two SNPs significantly increased the probability of being healthy, thus representing a protective factor against IHD (OR: 0.185; 95%CI: 0.440–0.770; *p* = 0.02), regardless of CV risk factors. The protective role of the rs5215_G/G genotype has been already demonstrated [18,19], while the role of rs1799983_T/T genotype is controversial in the literature. The presence of the T allele may associate with CV protection and reduced vascular stiffness [55], even if other evidence has demonstrated a predisposing role to vascular stiffness [56] and IHD [57] through the reduced production of NO [58]. Other evidence showed no relationship between this SNP and IHD [59]. However, we speculate that, beyond the individual role of each genetic variant, the co-presence of the two polymorphisms may cause a mutual, positive influence between eNOS and the K_ATP_ channel, which may promote a protective vascular effect, preserving coronary circulation, both in the epicardial district and microcirculation, from atherosclerotic damage and vasomotor tone impairment, and reducing the risk of developing IHD.

In conclusion, the main findings of the study are the following:

(1) The association of rs5215_G/G of KCNJ11 and rs1799983_T/T of NOS3 is more prevalent in healthy subjects compared with CAD and CMD patients.
(2) The association of rs5215_G/G of KCNJ11 and rs1799983_T/T of NOS3 may potentially represent a protective factor against IHD, regardless of traditional CV risk factors.

Our study has several limitations. It reports on the experience of a single center. The study population is mainly characterized by Caucasian patients, and it did not consider other ethnicities for which the genetic background may be different. The study was conducted based on genotype, and the tissue expression of proteins, their function, eventual post-translational modification, and change in coronary molecular microenvironment were not studied. We did not use inhibitors of the NO pathway to study the variation of NO metabolites according to NOS3 genetic variants. Our study focuses only on a few SNPs of two proteins involved in CBF regulation, missing an exhaustive investigation of other mechanisms involved in CBF and IHD.

## 4. Materials and Methods

In the present prospective, observational, single-center study, a total of 468 consecutive patients admitted to the Department of Clinical Internal, Anesthesiology and Cardiovascular Sciences of Sapienza University of Rome were enrolled from 2014 to 2021. According to published guidelines [1,2,4], all patients enrolled had an indication to perform CAG due to suspected IHD. Inclusion criteria for the study were: (i) written and signed informed consent; (ii) age ≥ 18 years; and (iii) documented and/or suspected acute and/or chronic myocardial ischemia requiring diagnostic investigation with CAG, according to Guidelines [1,2,4]. Exclusion criteria of the study were: (i) concomitant genetic disorders predisposing to IHD; (ii) documented and/or suspected genetic and/or acquired cardiomyopathy; (iii) end stage renal disease; (iv) protocol deviation; or (v) patient’s decision to drop-out from the study.

The following baseline features were collected in a dedicated Excel database: age; gender; body mass index (BMI); CV risk factors (i.e., arterial hypertension, diabetes mellitus, dyslipidemias, smoking habit, familial history of IHD); presence of ischemic EKG abnormalities; previous cardiological history (i.e., previous myocardial ischemia, presence of pacemaker and implantable cardioverter defibrillator, atrial fibrillation); presence of chronic kidney disease; chronic obstructive pulmonary disease; history of cancer; clinical presentation at admission (i.e., ST-segment elevation myocardial infarction (STEMI), non-ST-segment elevation myocardial infarction (NSTEMI), unstable angina (UA), stable angina (SA), asymptomatic presentation); and baseline echocardiographic parameters (left ventricular end diastolic diameter, interventricular septum thickness, posterior wall thickness, LVEF).

All patients approved and signed the informed consent. All study procedures were performed according to the 2013 Declaration of Helsinki. The present protocol (RIF. CE. 5261) was approved by the Ethical Committee of the Policlinico Umberto I hospital of Rome.

### 4.1. Study Protocol

Patients underwent a comprehensive CV evaluation. Before the CAG, a physical examination, 12-lead EKG, and transthoracic echocardiogram were performed. A peripheral blood sample was taken to perform genetic analysis.

The CAG was performed using radial or femoral artery access following Judkins approach. Patients with epicardial atherosclerotic obstructive lesions were treated according to Guidelines [60]. In patients without epicardial obstructive atherosclerotic disease, intracoronary (IC) functional tests, using Doppler-based and thermodilution techniques were performed to assess the presence of endothelial-dependent and endothelial-independent CMD, as suggested by Guidelines and consensus documents [2,10,61,62]. In particular, endothelial-independent microvascular function was assessed through the infusion of 30–60 μg of adenosine into the right coronary artery and 60–120 μg of adenosine into the left coronary artery [61]. Endothelial-dependent microvascular function was studied through the IC acetylcholine sequential infusion of 18.2 μg/mL at 1 mL/min for 2 min followed by 2 mL/min for 2 min into the left coronary artery and of half the dose or rate into the right coronary artery [61]. IC functional tests allowed the estimation of the coronary flow reserve (CFR) and index of microvascular resistance (IMR) [61].

**Figure 1:**
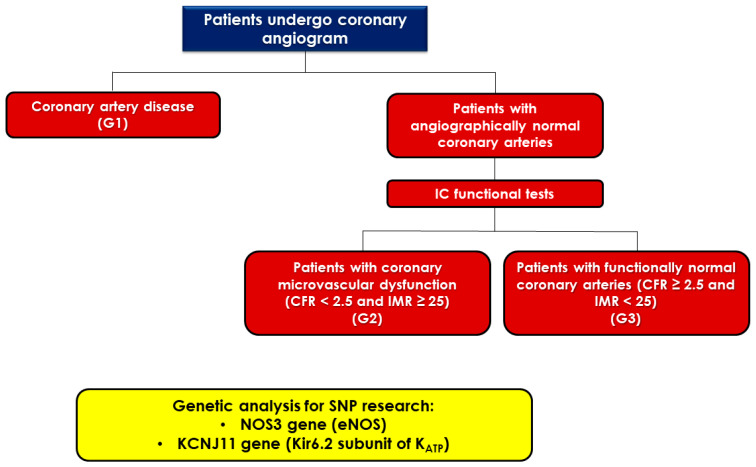
Flow chart representing study design. Patients underwent a coronary angiogram (CAG) due to suspected or confirmed acute or chronic ischemic heart disease. They were divided into 3 groups according to the CAG results: G1 (patients with coronary artery disease), G2 (patients with coronary microvascular dysfunction), and G3 (patients with angiographically and functionally normal coronary arteries). A peripheral blood sample was taken from each patient for genetic analysis and single nucleotide polymorphisms of KCNJ11 and NOS 3 research. IC: intracoronary; CFR: coronary flow reserve; IMR: index of microvascular resistance; SNPs: single nucleotide polymorphisms; NOS3: nitric oxide synthase 3; eNOS: endothelial nitric oxide synthase; KCNJ11: potassium inwardly rectifying channel subfamily J member 11; K_ATP_: ATP sensitive potassium channels.

Based on diagnostic CAG and IC functional tests, the study population was divided in three groups (Figure 1):

1. G1: patients affected by significant coronary artery disease (CAD), defined by the presence of a stenosis ≥ 50% of the epicardial vessel lumen.
2. G2: patients affected by CMD, defined by the presence of a CFR < 2.5 and IMR ≥ 25, assessed through IC functional tests and under conditions of angiographically normal coronary arteries.
3. G3: patients whose CAG and IC functional tests show angiographically and functionally normal coronary arteries (CFR ≥ 2.5 and IMR < 25 after infusion of acetylcholine and adenosine).

Genetic analysis was conducted according to international guidelines [63]. Ethylenediaminetetraacetic acid (EDTA) peripheral whole blood sample was taken from patients. A hemostatic tourniquet was placed on the upper right arm of the patient in order to better display veins. Skin was sanitized with hydrogen peroxide, a sterile needle was inserted into the vein and linked to a 10 mL container tube. When the blood collection was completed, the hemostatic tourniquet and the needle were removed and a bandage was placed on the puncture site, applying pressure for few minutes to stop the bleeding. The EDTA tubes obtained were sent to the Department of Biomedical Sciences and Clinical Oncology and Oncogenomic research Centre, “Aldo Moro” University of Bari, where they were stored at −80° until DNA extraction and analysis. Based on the literature and our previous results [18,19,52,64,65], we analyzed SNP rs5215 of the KCNJ11 gene, encoding for the Kir6.2 subunit of the K_ATP_ channel and located on chromosome 11p15.1, and SNP rs1799983 of the nitric oxide synthase 3 (NOS3) gene, located on chromosome 7q35-7q36 and encoding for eNOS.

Genomic DNA was isolated from leucocytes using the ISOLATE II genomic DNA kit (Bioline Reagents Ltd., Meridian Bioscience, London, UK), an ionic exchange column-based kit, according to the manufacturer’s instructions. The SNPs were determined by a standard polymerase chain reaction (PCR) amplification using the HotStarTaq Master Mix kit (QIAGEN Inc., Valencia, CA, USA) in a GeneAmp PCR System 9700 (Life Technologies, Carlsbad, CA, USA) as follows: a first step of DNA denaturation at 95 °C for 15 min, 32 cycles of 94 °C for 30 s, 58 °C for 30 s, and 72 °C for 1 min, and a final extension step at 72 °C for 10 min. The primer used for SNP rs5215 of KCNJ11 was 5′-TGGACATCCCCATGGAGAAC-3′. Primers for rs1799983 of the NOS3 gene (F5′-CATGAGGCTCAGCCCCAGAAC-3′ and R5′-AGTCAATCCCTTTGGTGCTCAC-3′) were selected from the NOS3 Ensemble sequence database (Ensembl: ENSG00000164867) and designed using the free web-based application Primer3Plus (https://www.bioinformatics.nl/cgi-bin/primer3plus/primer3plus.cgi (accessed on 10 September 2021). Direct sequencing analyses were performed on both forward and reverse strands with the same pair of primers using the Big Dye Terminator v3.1 Cycle Sequencing kit (Applied Biosystem, Waltham, MA, USA) and run on a 3500 Genetic Analyzer (Thermo Fisher Scientific Inc., Waltham, MA, USA).

### 4.2. Definition of Cardiovascular Risk Factors, Coronary Artery Disease (CAD), and Coronary Microvascular Dysfunction (CMD)

CV risk factors were defined as follows: diabetes mellitus was present in patients under treatment with antidiabetic drugs or with fasting glucose values ≥ 126 mg/dL in three consecutive measurements away from meals and/or in the presence of HbA1c ≥ 7% (≥53 mmol/mol); smoking habit was present if, at the enrolment time, patients smoked tobacco or suspended it less than 12 months ago; arterial hypertension was present if patients were treated with anti-hypertensive drugs or if an arterial pressure ≥ 140 mmHg and ≥90 mmHg for systolic and diastolic pressure, respectively, was documented during three consecutive measurements. Hypercholesterolemia was present if patients were on treatment with cholesterol-lowering drugs or if they had a low-density lipoprotein (LDL) value ≥ 55 mg/dL when considered at very high risk or in secondary prevention, ≥70 mg/dL when at high risk, ≥100 mg/dL when at moderate risk, and ≥116 mg/dL when at low risk. Obesity was present if patients had a BMI ≥ 30 kg/m^2^; familial history for CV diseases was present if patients had a first-degree relative who manifested IHD before the age of 60.

CAD was defined, according to guidelines [1,2,3,4], by the presence of an atherosclerotic plaque causing a stenosis ≥ 50% of the epicardial coronary artery diameter at the CAG [1,2,3,4,21]. CMD was defined by CFR values < 2.5 and IMR ≥ 25, after IC functional tests, in the absence of epicardial atherosclerotic plaque ≥ 50% of vessel diameter at the CAG [21].

### 4.3. Statistical Analysis

The sample size was calculated assuming a 15% prevalence of normal microvascular and macrovascular coronary findings in unselected patients undergoing a CAG. To enable the computation of two-sided 95% confidence intervals for such prevalence estimates ranging between −5.0 and +5.0%, we estimated a sample size of at least 150 patients. The Kolmogorov–Smirnov test was used for the assessment of normal distribution of variables. Categorical variables were expressed as number and percentages; for continuous variables, the mean and standard deviation or the median and first and third quartiles were used, as needed. Baseline demographic and clinical characteristics were presented in table format. Comparisons among variables were made using the Student’s *t*-test for normally distributed continuous variables, whereas categorical variables were compared using the χ^2^ test and the Fisher exact test. The Mann–Whitney U test and the Kruskal–Wallis test was used to assess the differences between variables with a non-normal distribution. For all tests, a *p*-value less than 0.05 was considered statistically significant. The observed numbers of each genotype were compared with those expected for a population in Hardy–Weinberg equilibrium using a free web-based application. To estimate the association between genetic polymorphisms, CV risk factors, and IHD by logistic regression analysis, the odds ratios (ORs) and their 95% confidence intervals (95%CIs) were calculated. Univariate and multivariate analyses were performed, and all the variables with a significant association (*p*-value < 0.10) in the univariate analysis were included in the multivariate analysis. The statistical analysis was performed using SPSS version 27.0 for Mac (IBM Software, Inc., Armonk, NY, USA).

## 5. Conclusions

Our results suggest the potential role of genetic variants for the genes encoding the K_ATP_ channel and eNOS in IHD susceptibility, beyond traditional CV risk factors. In particular, this is the first study to discover that the co-presence of two SNPs, rs5215_G/G of KCNJ11 and rs1799983_T/T of NOS3, may be potentially protective against IHD, regardless of CV risk factors. This result allows the hypothesis that eNOS and K_ATP_ channels may interact and influence each other in the regulation of CBF, determining a potentially protective effect both on epicardial arteries and microcirculation. The presence of these two SNPs might suggest a positive crosstalk between eNOS and K_ATP_ channels on the complex network of coronary circulation, hypothetically counteracting both atherosclerotic disease and vasomotor tone dysregulation, thus providing protection against IHD. Our results support the hypothesis that genetics may represent a primary mechanism in IHD susceptibility, shedding light on potential targets for gene therapy against IHD. Although these results may be potentially interesting, they should be confirmed in a wider population. Moreover, the real impact of genetic variants on proteins involved in CBF regulation and on the coronary microenvironment has to be assessed.

## Figures and Tables

**Table 1:** Baseline features of study population and differences according to each group (G1: patients with coronary artery disease; G2: patients with coronary microvascular dysfunction; G3: healthy patients). Continuous variables are expressed as the median and first and third quartiles [Q1;Q3]. For continuous variables, the overall difference among groups was calculated using the Kruskal–Wallis test and the Mann–Whitney U test was used for testing differences among groups. The χ^2^ test was used for testing differences in categorical variables.

Parameters	G1 (N = 328)	G2 (N = 68)	G3 (N = 66)	*p*-Value	Post-hoc *p*-Value
**Age, years [Q1;Q3]**	69 [60;78]	65 [56;71]	60.5 [54.8;69]	<0.001	G1-G2 < 0.001; G1-G3 < 0.001
**Male,** **n (%)**	252 (76.8%)	30 (44.1%)	31 (47%)	<0.001	G1-G2 < 0.001; G1-G3 < 0.001
**BMI [Q1;Q3]**	26 [24.5;27.2]	26 [24.4;27.8]	26 [24.3;26.5]	0.783	G1-G2 = 1; G1-G3 = 1
**Arterial hypertension, n (%)**	276 (84.1%)	67 (98.5%)	66 (100%)	<0.001	G1-G2 < 0.001; G1-G3 < 0.001
**Diabetes mellitus,** **n (%)**	103 (31.4%)	12 (17.6%)	13 (19.7%)	0.020	G1-G2 = 0.027; G1-G3 = 0.037
**Dyslipidemia,** **n (%)**	187 (57%)	29 (42.6%)	24 (36.4%)	0.002	G1-G2 = 0.033; G1-G3 = 0.002
**Smoking habit,** **n (%)**	164 (50%)	19 (27.9%)	23 (34.8%)	<0.001	G1-G2 = 0.001; G1-G3 = 0.017
**Family history of IHD, n (%)**	140 (42.7%)	24 (35.3%)	21 (31.8%)	0.178	G1-G2 = 0.282; G1-G3 = 0.131
**LVEF, % [Q1;Q3]**	50 [40;55]	55 [50;56.5]	55 [51.5;56.5]	<0.001	G1-G2 < 0.001; G1-G3 < 0.001

BMI: body mass index; IHD: ischemic heart disease; LVEF: left ventricular ejection fraction.

**Table 2:** Distribution of each single nucleotide polymorphism (SNP) of KCNJ11 and NOS3 genes and their interaction, across study groups (G1 = coronary artery disease; G2 = coronary microvascular dysfunction; G3: healthy patients). The χ^2^ test was used for testing differences in categorical variables.

Protein/Gene: SNP	G1 (N = 328)	G2 (N = 68)	G3 (N = 66)	*p*-Value	Post-Hoc *p*-Value
**Kir6.2/KCNJ11: rs5215_A/A, n (%)**	142 (43.3%)	35 (51.5%)	31 (47%)	0.440	G1-G2 = 0.230; G1-G3 = 0.590; G2-G3 = 0.602
**Kir6.2/KCNJ11: rs5215_G/A, n (%)**	145 (44.2%)	27 (39.7%)	23 (34.8%)	0.337	G1-G2 = 0.591; G1-G3 = 0.174; G2-G3 = 0.561
**Kir6.2/KCNJ11: rs5215_G/G, n (%)**	41 (12.5%)	6 (8.8%)	12 (18.2%)	0.258	G1-G2 = 0.536; G1-G3 = 0.236; G2-G3 = 0.112
**eNOS/NOS3: rs1799983_G/G, n (%)**	68 (45.3%)	17 (37.8%)	20 (48.8%)	0.557	G1-G2 = 0.396; G1-G3 = 0.726; G2-G3 = 0.303
**eNOS/NOS3: rs1799983_T/T, n (%)**	58 (38.7%)	16 (35.6%)	18 (43.9%)	0.724	G1-G2 = 0.730; G1-G3 = 0.591; G2-G3 = 0.429
**eNOS/NOS3: rs1799983_G/T, n (%)**	24 (16%)	12 (26.7%)	3 (7.3%)	0.052	G1-G2 = 0.126; G1-G3 = 0.209; G2-G3 = 0.018
**Kir6.2/KCNJ11: rs5215_A/A x eNOS/NOS3: rs1799983_G/G, n (%)**	32 (12.8%)	11 (19.6%)	8 (15.1%)	0.407	G1-G2 = 0.202; G1-G3 = 0.657; G2-G3 = 0.532
**Kir6.2/KCNJ11: rs5215_A/A x eNOS/NOS3: rs1799983_T/T, n (%)**	21 (8.4%)	5 (8.9%)	9 (17%)	0.156	G1-G2 = 0.797; G1-G3 = 0.074; G2-G3 = 0.209
**Kir6.2/KCNJ11: rs5215_A/A x eNOS/NOS3: rs1799983_G/T, n (%)**	21 (8.4%)	5 (8.9%)	9 (17%)	0.156	G1-G2 = 0.797; G1-G3 = 0.074; G2-G3 = 0.209
**Kir6.2/KCNJ11: rs5215_G/G x eNOS/NOS3: rs1799983_G/G, n (%)**	8 (2.6%)	3 (4.5%)	2 (3.2%)	0.722	G1-G2 = 0.426; G1-G3 = 0.684; G2-G3 = 0.699
**Kir6.2/KCNJ11: rs5215_G/G x eNOS/NOS3: rs1799983_T/T, n (%)**	6 (2%)	0 (0%)	5 (7.9%)	0.008	G1-G2 = 0.246; G1-G3 = 0.026; G2-G3 = 0.019
**Kir6.2/KCNJ11: rs5215_G/G x eNOS/NOS3: rs1799983_G/T, n (%)**	3 (1%)	2 (3%)	2 (3.2%)	0.284	G1-G2 = 0.199; G1-G3 = 0.205; G2-G3 = 0.950
**Kir6.2/KCNJ11: rs5215_G/A x eNOS/NOS3: rs1799983: G/G, n (%)**	28 (11.1%)	3 (5.2%)	10 (17.5%)	0.109	G1-G2 = 0.174; G1-G3 = 0.185; G2-G3 = 0.036
**Kir6.2/KCNJ11: rs5215_G/A x eNOS/NOS3: rs1799983_T/T, n (%)**	31 (12.3%)	11 (19%)	4 87%)	0.151	G1-G2 = 0.181; G1-G3 = 0.355; G2-G3 = 0.057
**Kir6.2/KCNJ11: rs5215_G/A x eNOS/NOS3: rs1799983_G/T, n (%)**	10 (4%)	3 (5.2%)	0 (0%)	0.262	G1-G2 = 0.680; G1-G3 = 0.218; G2-G3 = 0.082

Kir: inward-rectifier potassium channels; KCNJ11: Potassium inwardly rectifying channel subfamily J member 11; eNOS: endothelial nitric oxide synthase; NOS3: nitric oxide synthase 3.

**Table 3:** Univariable and multivariable binary regression analysis for risk factors and single nucleotide polymorphisms (SNPs) of KCNJ11 and NOS3 genes with regard to the prediction of coronary artery disease (CAD).

	Univariable			Multivariable		
	OR	95% CI	*p*-Value	OR	95% CI	*p*-Value
**Age**	1.047	1.029–1.064	<0.001	1.063	1.040–1.086	<0.001
**Male gender**	3.968	2.592–6.075	<0.001	5.248	3.148–8.751	<0.001
**Arterial hypertension**	0.040	0.005–0.292	0.002	0.041	0.006–0.309	0.002
**Diabetes mellitus**	1.996	1.219–3.268	0.006	1.646	0.932–2.908	0.086
**Dyslipidemia**	2.027	1.346–3.053	<0.001	1.986	1.221–3.229	0.006
**Smoking habit**	2.190	1.433–3.348	<0.001	2.192	1.312–3.660	0.003
**Familial history of IHD**	1.473	0.968–2.242	0.071	1.757	1.070–2.884	0.026
**Kir6.2/KCNJ11: rs5215_A/A**	0.787	0.526–1.177	0.243			
**Kir6.2/KCNJ11: rs5215_G/A**	0.751	0.497–1.135	0.174			
**Kir6.2/KCNJ11: rs5215_G/G**	0.921	0.508–1.669	0.785			
**eNOS/NOS3: rs1799983_G/G**	0.911	0.534–1.554	0.731			
**eNOS/NOS3: rs1799983_T/T**	0.964	0.560–1.660	0.895			
**eNOS/NOS3: rs1799983_G/T**	0.902	0.444–1.829	0.774			
**Kir6.2/KCNJ11: rs5215_G/G x eNOS/NOS3 rs1799983_T/T**	0.503	0.151–1.680	0.264			

IHD: ischemic heart disease; Kir: inward-rectifier potassium channels; KCNJ11: Potassium inwardly rectifying channel subfamily J member 11; eNOS: endothelial nitric oxide synthase; NOS3: nitric oxide synthase 3; OR: odds ratio; CI: confidence interval.

**Table 4:** Univariable and multivariable binary regression analysis for risk factors and single nucleotide polymorphisms (SNP) of KCNJ11 and NOS3 genes with regard to the prediction of coronary microvascular dysfunction (CMD).

	Univariable			Multivariable		
	OR	95% CI	*p*-Value	OR	95% CI	*p*-Value
**Age**	0.970	0.950–0.990	0.003	0.961	0.933–0.990	0.009
**Male gender**	0.310	0.183–0.524	<0.001	0.288	0.139–0.595	<0.001
**Arterial hypertension**	10.187	1.384–74.971	0.023	9.372	1.453–81.329	0.037
**Diabetes mellitus**	0.514	0.265–0.994	0.048	0.533	0.208–1.366	0.190
**Dyslipidemia**	0.645	0.384–1.085	0.098	0.736	0.356–1.519	0.407
**Smoking habit**	0.429	0.244–0.756	0.003	0.429	0.191–0.967	0.041
**Familial history of IHD**	0.789	0.462–1.350	0.387			
**Kir6.2/KCNJ11: rs5215_A/A**	1.355	0.809–2.269	0.248			
**Kir6.2/KCNJ11: rs5215_G/A**	0.886	0.524–1.498	0.651			
**Kir6.2/KCNJ11: rs5215_G/G**	0.623	0.257–1.511	0.295			
**eNOS/NOS3: rs1799983_G/G**	0.711	0.365–1.384	0.315			
**eNOS/NOS3: rs1799983_T/T**	0.835	0.425–1.641	0.601			
**eNOS/NOS3: rs1799983_G/T**	0.453	0.208–0.984	0.045	0.768	0.157–0.962	0.121
**Kir6.2/KCNJ11: rs5215_G/G x eNOS/NOS3 rs1799983_T/T**	0.490	0.062–3.862	0.499			

IHD: ischemic heart disease; Kir: inward-rectifier potassium channels; KCNJ11: Potassium inwardly rectifying channel subfamily J member 11; eNOS: endothelial nitric oxide synthase; NOS3: nitric oxide synthase 3; OR: odds ratio; CI: confidence interval.

**Table 5:** Univariable and multivariable binary regression analysis for risk factors and single nucleotide polymorphisms of KCNJ11 and NOS3 genes with regard to the prediction of ischemic heart disease (IHD). The interaction between Kir6.2/KCNJ11: rs5215_G/G and eNOS/NOS3: rs1799983_T/T is associated with a significantly lower risk of IHD.

	Univariable			Multivariable		
	OR	95% CI	*p*-Value	OR	95% CI	*p*-Value
Age	1.047	1.029–1.064	<0.001	1.047	1.015–1.080	0.003
Male gender	2.793	1.644–4.745	<0.001	3.835	1.777–8.275	<0.001
Arterial hypertension	0.418	0.146–1.195	0.104			
Diabetes mellitus	1.668	0.876–3.178	0.119			
Dyslipidemia	2.1	1.225–3.6	0.007	1.158	0.554–2.422	0.696
Smoking habit	1.606	0.933–2.766	0.087	1.826	0.785–4.248	0.162
Familial history of IHD	1.515	0.869–2.639	0.143			
Kir6.2/KCNJ11: rs5215_A/A	0.913	0.541–1.539	0.731			
Kir6.2/KCNJ11: rs5215_G/A	1.436	0.833–2.473	0.193			
Kir6.2/KCNJ11: rs5215_G/G	0.606	0.302–1.215	0.158			
eNOS/NOS 3: rs1799983_G/G	0.811	0.413–1.593	0.544			
eNOS/NOS3: rs1799983_T/T	0.781	0.395–1.544	0.478			
eNOS/NOS3: rs1799983_G/T	2.868	0.838–9.810	0.093	2.623	0.728–9.442	0.140
Kir6.2/KCNJ11: rs5215_G/G x eNOS/NOS3 rs1799983_T/T	0.191	0.056–0.645	0.008	0.185	0.440–0.770	0.020

IHD: ischemic heart disease; Kir: inward-rectifier potassium channels; KCNJ11: Potassium inwardly rectifying channel subfamily J member 11; eNOS: endothelial nitric oxide synthase; NOS3: nitric oxide synthase 3; OR: odds ratio; CI: confidence interval.

## Data Availability

The data presented in this study are available on request from the corresponding author.
